# Ribonuclease H1-dependent hepatotoxicity caused by locked nucleic acid-modified gapmer antisense oligonucleotides

**DOI:** 10.1038/srep30377

**Published:** 2016-07-27

**Authors:** Takeshi Kasuya, Shin-ichiro Hori, Ayahisa Watanabe, Mado Nakajima, Yoshinari Gahara, Masatomo Rokushima, Toru Yanagimoto, Akira Kugimiya

**Affiliations:** 1 Discovery Research Laboratories for Innovative Frontier Medicines, Shionogi & Co., LTd., Osaka, 561-0825, JAPAN; 2 Research Laboratory for Development, Shionogi & Co., LTd., Osaka, 561-0825, JAPAN

## Abstract

Gapmer antisense oligonucleotides cleave target RNA effectively *in vivo*, and is considered as promising therapeutics. Especially, gapmers modified with locked nucleic acid (LNA) shows potent knockdown activity; however, they also cause hepatotoxic side effects. For developing safe and effective gapmer drugs, a deeper understanding of the mechanisms of hepatotoxicity is required. Here, we investigated the cause of hepatotoxicity derived from LNA-modified gapmers. Chemical modification of gapmer’s gap region completely suppressed both knockdown activity and hepatotoxicity, indicating that the root cause of hepatotoxicity is related to intracellular gapmer activity. Gene silencing of hepatic ribonuclease H1 (RNaseH1), which catalyses gapmer-mediated RNA knockdown, strongly supressed hepatotoxic effects. Small interfering RNA (siRNA)-mediated knockdown of a target mRNA did not result in any hepatotoxic effects, while the gapmer targeting the same position on mRNA as does the siRNA showed acute toxicity. Microarray analysis revealed that several pre-mRNAs containing a sequence similar to the gapmer target were also knocked down. These results suggest that hepatotoxicity of LNA gapmer is caused by RNAseH1 activity, presumably because of off-target cleavage of RNAs inside nuclei.

The 5′ and 3′ end-modified antisense oligonucleotide (ASO), gapmer, is promising therapeutic agent which can modulate target RNA expression^1^,^2^. Various types of chemical modification of ASOs have been examined to enhance nuclease resistance, to improve stability of ASO–RNA hybrids, and to increase RNA-manipulating effects. The initially developed gapmer was modified with 2′-methoxyethyl (MOE) nucleotides and its phosphodiester backbone was fully substituted for phosphorothioate (PS) bonds^3^. Those modifications concurrently enhanced the gapmer’s RNA knockdown ability. Since then, locked nucleic acid (LNA) has developed for further improve the biological effects of gapmers^4^,^5^. Modification with LNA at the 5′- and 3′ ends of gapmer considerably improved the thermodynamic stability of RNA–gapmer duplex, as well as nuclease resistance^6^. Generally, LNA gapmer shows higher *in vivo* RNA silencing activity than do gapmers modified with MOE, at same dose^7^.

Gapmer causes target-RNA knockdown, using the function of intracellular ribonuclease H1 (RNAseH1). RNAseH1 is ubiquitously expressed and localized in the nucleus and mitochondria. It recognizes RNA–DNA duplexes and catalyses non-specific cleavage of RNA strand. It is considered to play a pivotal role for DNA replication and repair by hydrolysing phosphodiester bond between DNA-RNA hybridized sections^8^,^9^,^10^. Modification of gapmer with LNA is considered to facilitate efficient target-RNA knockdown by enhancing hybridization and its stability of RNA–gapmer duplexes inside the nucleus. Overexpression of RNaseH1 can increase the knockdown effect of gapmer both *in vitro* (cultured cells) and *in vivo* (mice)^11^.

Recently, Food and Drug Administration in United States approved a first gapmer drug, mipomersen, for the treatment of familial homozygous hyperchoresterolemia. Mipomersen is MOE-modified gapmer targeting apolipoprotein B-100 mRNA in human liver^12^,^13^,^14^. It shows significant therapeutic effect by decreasing plasma low-density apolipoprotein for 25–35% in patients. However, mipomersen also showed important side effect: 10–20% of patients suffered liver damage^15^. More recently, Committee for Medicinal Products for Human Use of European Medicines Agency decided to refuse its approval because of the risk of irreversible liver damage^16^. LNA-modified gapmers show a stronger knockdown effect than do MOE-modified gapmers; therefore, not only therapeutic effect but also the side effects are likely to be more intense. In fact, several reports have shown that LNA gapmers lead to severe toxicity^7^,^17^,^18^. For example, a single administration of several LNA gapmers to mice resulted in a significant increase of clinical markers for hepatotoxicity, serum alanine aminotransferase (ALT) and aspartate aminotransferase (AST), whereas the gapmers modified by MOE showed no or low hepatotoxicity, albeit with a weaker knockdown effect at same dose^7^. Although other types of chemically modified nucleotides have been developed to reduce hepatotoxicity without decreasing the knockdown effect, those modifications exhibited similar or weaker knockdown effects and weaker hepatotoxicity than those modified with LNA^19^,^20^,^21^,^22^,^23^. Those investigations have indicated that varying the chemical modification can modulate RNA knockdown activity, but risk of hepatotoxicity cannot be eliminated. So far several reports have shown sequence motifs and/or modifications that could enhance toxicity^24^,^25^, and the transcription profiles of toxic gapmers have been analysed using microarray analysis^26^. However, the common mechanisms of gapmer toxicity had not been elucidated. Recently, Burel *et al.* suggested that the hepatotoxicity would be mediated by promiscuous knockdown^27^. They found that the expression levels of many unintended transcripts, especially long pre-mRNAs, were downregulated by administration of toxic gapmers.

The following four hypotheses can be considered in regard to the mechanism of hepatotoxicity caused by gapmers: 1. biochemical/biophysical toxicity derived from non-natural oligonucleotide(s); 2. innate immune responses caused by toll-like receptors (TLRs); 3. on-target knockdown-related dysfunction; and 4. off-target knockdown of essential RNAs. Considering recent findings by Burel *et al.*, the off-target effect would be an important factor; however there is a possibility that the other factors are involved in gapmer’s hepatotoxicity.

The aim of our study was to address the major mechanism(s) of the liver toxicity of gapmers. We herein show that chemical modification of the gap region of these LNA gapmers critically suppresses hepatotoxic effect. Furthermore, small interfering RNA (siRNA)-mediated silencing of RNaseH1 successfully reduces gapmer hepatotoxicity, while the knockdown of RNaseH2a shows no significant effects. Gapmer-mediated knockdown results in severe hepatotoxicity, but siRNA targeting the same position on the target mRNA does not. Microarray analysis reveals that several pre-mRNAs, which has a sequence similar to that of the gapmer target, are knocked down after gapmer administration. We also find that these pre-mRNAs contain presumptive target sequence in their introns, which have 5 or fewer mismatches to the parental target sequence. This unintended knockdown is not occurred by gap-modified LNA gapmers, as well as siRNA. Moreover, silencing of RNaseH1 in liver critically suppressed the reduction of those pre-mRNAs. Our results suggest that the hepatotoxicity is derived from the knockdown activity, and arises because of off-target RNA cleavage by the low-specificity of LNA gapmer.

## Results

### *In vivo* hepatotoxicity of gapmers and ‘non-gapmers’

We examined the mechanisms of hepatotoxicity caused by gapmers, largely utilizing two LNA gapmers. One targets the glucocorticoid receptor (GR; also known as Nr3c1). GR is ubiquitously expressed and regulates various cellular activities in response to ligand binding^28^. The GR gapmer was previously reported to bring about both a knockdown effect and potent liver toxicity *in vivo*, with a dramatic increase in plasma ALT/AST levels^17^. The other gapmer used here targets Acyl-CoA synthetase long-chain family member 1 (Acsl1). Acsl1 is expressed in liver and striated muscle, and plays an important role in lipid synthesis by catalysing the conversion of long-chain fatty acids to fatty acyl-CoA thioesters^29^. The Acsl1 gapmer used in this study significantly increases plasma ALT/AST levels within a few days after a single administration.

**Figure 1:**
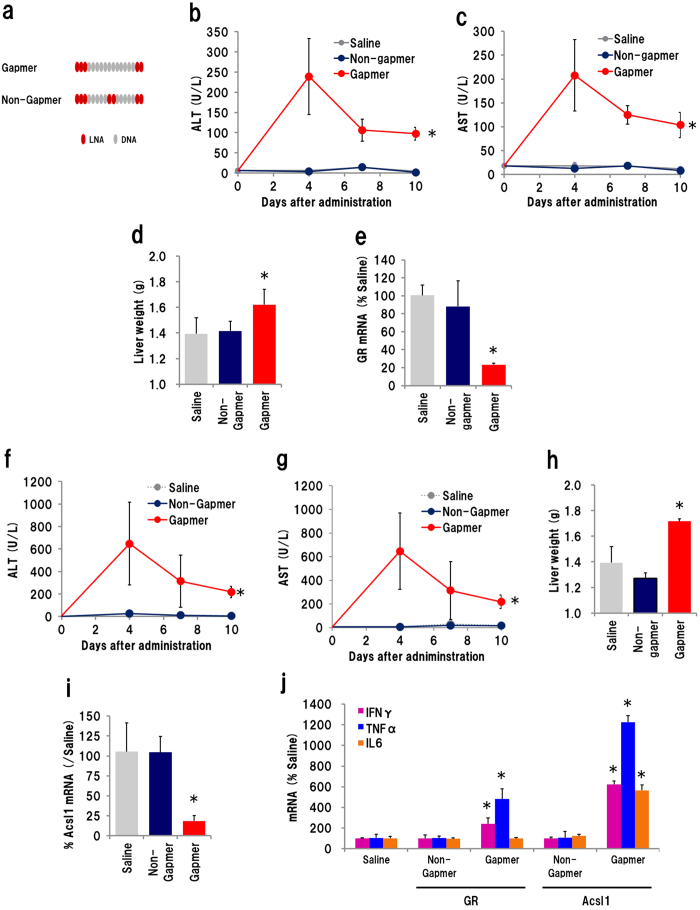
Hepatotoxicity of gapmer and ‘non-gapmer’. (**a**) Schematic illustration of a gapmer and non-gapmer. The non-gapmer was designed to reduce gapmer activity by substituting two naïve deoxyribonucleotides for LNA at the centre of the gap. Plasma ALT (**b**), AST (**c**) levels in mice received 10 mg/kg of GR ASO were analysed at 4, 7 and 10 days after administration. Whole liver weight (**d**) and *GR* mRNA level (**e**) were measured on day 10. The same experiments were performed with Acsl1 gapmer and non-gapmer (20 mg/kg): plasma ALT (**f**), AST (**g**), liver weight (**h**) *Acsl1* mRNA level (**i**), and mRNA level of IFNγ, TNFα, IL6 (**j**). (n = 4, mean ± S.D. *p < 0.05, Mann–Whitney *U* test, vs saline).

We first investigated the possibility of biophysical/biochemical toxicity. To examine whether a non-functional, gapmer-like ASO would show hepatotoxicity, we designed a ‘non-gapmer’ composed of the same nucleotide sequence but missing the ‘gap’ required for RNA knockdown (Fig. 1a). Mammalian RNaseH1 digests an RNA strand hybridized with an LNA gapmer by recognizing a gap of at least seven nucleotides located between LNA-modified 5′ and 3′ positions^30^,^31^,^32^. We expected that substitution of two unmodified DNA nucleotides in the centre of gap would impair the knockdown activity of the gapmer. First we examined the GR-targeting gapmer and non-gapmer. ASOs were administrated to mice, plasma was collected on days 3 and 7, and liver was isolated at 10 days after injection. In the mice that received the gapmer, the plasma ALT/AST levels were increased on day 3, and a significant ALT/AST increase remained until day 10. Meanwhile, mice that received the non-gapmer did not exhibit any significant increase in plasma ALT/AST (Fig. 1b,c). Increase in liver weight is one of major parameters for liver inflammation caused by gapmers^7^. The weight of whole liver isolated from gapmer-received mouse was 1.16× heavier than that from control mice (Fig. 1d). qRT-PCR analysis revealed that GR mRNA was reduced by 77% in the liver of gapmer-recipient mice only (Fig. 1e). The same experiment was also performed using ASOs targeting *Acsl1*. In this case, the plasma ALT/AST level of gapmer-recipient mice were significantly increased, and remained so until day 10, while those of non-gapmer recipients remained at background levels (Fig. 1f,g). Administration of the Acsl1 gapmer resulted in an enlarged mouse liver, weighing 1.23× more than that from the saline- or non-gapmer-recipient mice (Fig. 1h). Eighty-one percent of *Acsl1* mRNA was silenced by the Acsl1 gapmer but not by the non-gapmer (Fig. 1i). We also examined mRNA expression of inflammatory cytokines, IFNγ, TNFα and IL6. Those mRNA was increased in gapmer-treated group, but not in non-gapmer-treated ones (Fig. 1j). These results suggested that the hepatotoxicity caused by the GR and Acsl1 gapmers is related to the RNA knockdown activity, presumably *via* an RNaseH1-dependent RNA cleavage mechanism.

### Effect of RNaseH1-knockdown on gapmer-derived hepatotoxicity

Knockdown of RNase H1 in mouse liver was efficiently performed by *i*.*v*. injection of an siRNA–invivofectamine complex. The siRNA for ribonuclease H2a (RNaseH2a) was also used; because RNaseH2 is a member of the RNase H enzymes that hydrolyse the RNA strand of an RNA/DNA duplex, it might be related to gapmer knockdown/hepatotoxic activity^33^,^34^. There are three subunits of RNaseH2: subunit A (H2a) possesses core domain required for RNA cleavage^35^,^36^. On day 6 after siRNA–invivofectamine injection, *Rnase H1* and *RnaseH2a* mRNA expression was efficiently regulated at 37% and 26% of levels in saline-injected mice, respectively (Supplementary Fig. S1a,b). Significant mRNA knockdown continued until day 12. The plasma ALT/AST levels showed no significant increase over the indicated time period (Supplementary Fig. S1c,d). Western blotting showed that the expression of RNaseH1 protein was decreased to less than 50% until day 12 (Supplementary Fig. S1e). We also investigated the expression levels of inflammatory cytokines, IFNα and γ. The expression of *IFNα* mRNA was unchanged, while *IFNγ* was slightly increased by all three siRNA-invivofectamine complex (150~170%). This subtle increase at mRNA level was considered to be tolerable to perform knockdown experiments (Supplementary Fig. S1f). Additionally, we checked cytotoxicity of these siRNAs. Transfection of those siRNAs did not show any impact on cell viability *in vitro* (Supplementary Fig. S2a,b). These results indicated that siRNA–invivofectamine complex achieved efficient gene silencing without causing hepatotoxic side effects.

**Figure 2:**
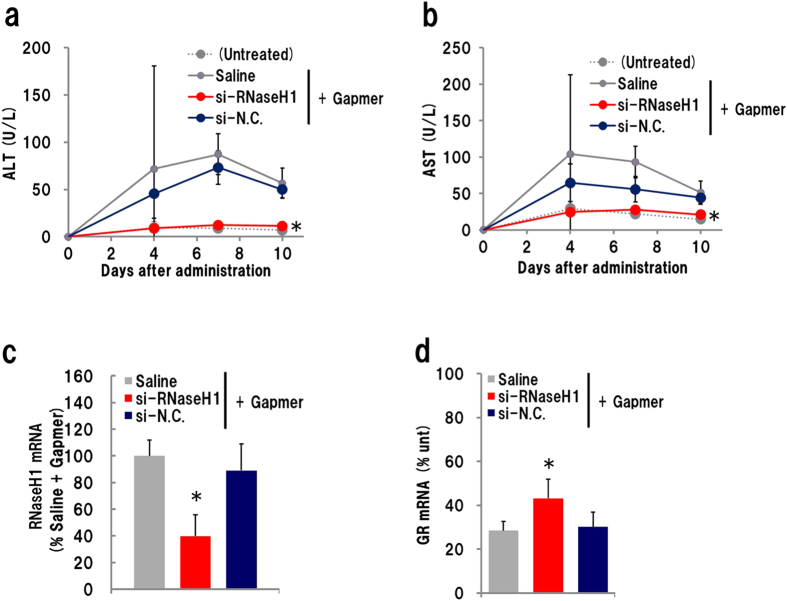
Effect of RNaseH1 knockdown for GR gapmer-derived hepatotoxicity. Mice were administrated an siRNA–invivofectamine complex 2 days prior to *s*.*c*. injection of 10 mg/kg gapmer. Plasma was collected 4, 7 and 10 days later, and mouse liver was isolated on day 10. (**a**) Plasma ALT level; (**b**) plasma AST level; (**c**) Expression level of *RnaseH1* and (**d**) *GR* mRNA in liver (n = 4, mean ± S.D., *p < 0.05, Mann–Whitney *U* test, vs saline + gapmer).

We investigated the effect of *in vivo* knockdown of RNaseH1 on gapmer-derived hepatotoxicity in combination with siRNA and gapmer administration. The GR gapmer was injected into mice that also received an *i*.*v*. administration of siRNA–invivofectamine complex two days before gapmer administration. Plasma ALT/AST levels were monitored. Surprisingly, plasma ALT/AST levels in mice that received si-RNAseH1 were unchanged, whereas those in mice pre-treated with saline or si-N.C. increased significantly (Fig. 2a,b). This suggested that siRNA-mediated knockdown of RNaseH1 completely suppressed the GR gapmer-derived hepatotoxicity. qRT-PCR showed that approximately 40% of *Rnaseh1* mRNA expression was abolished in the si-RNaseH1-pretreated group on day 10 (Fig. 2c). Administration of the gapmer showed a significant knockdown effect in each group, but it was slightly lower (approximately 10%) in si-RNaseH1-administered mice than in the mice treated with a different siRNA.

**Figure 3:**
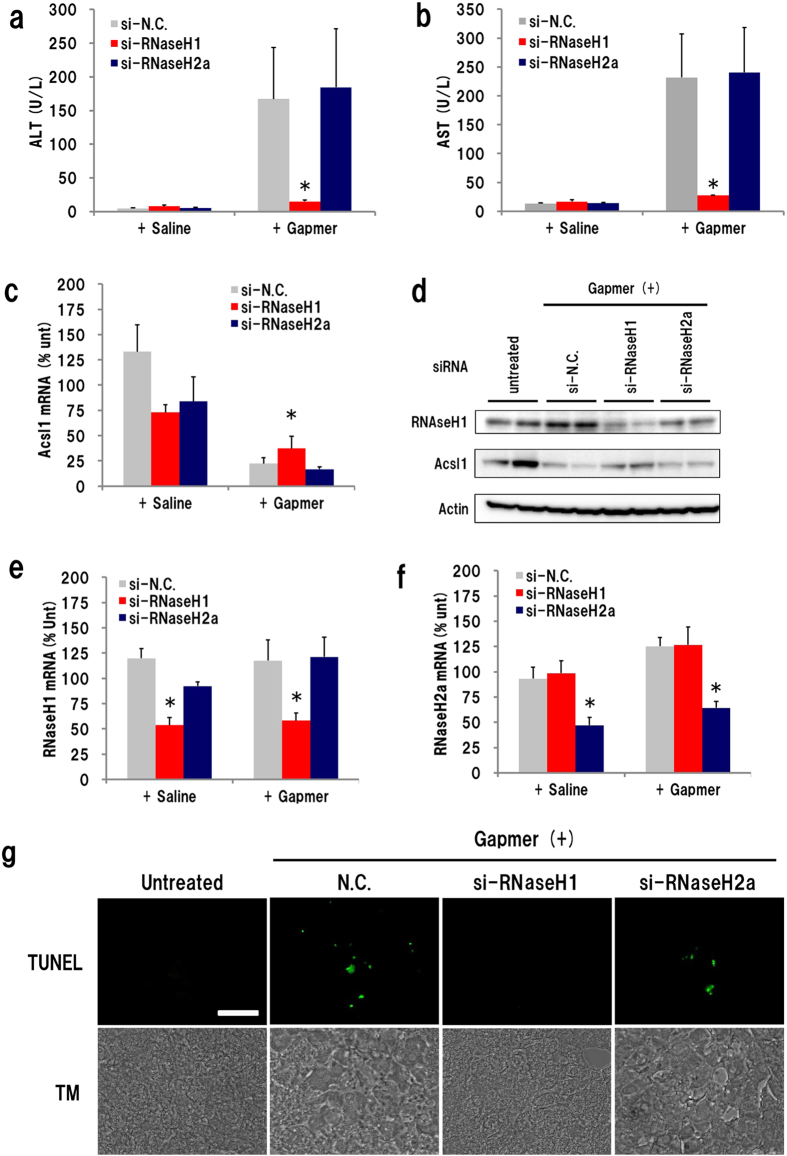
Effect of RNaseH1 knockdown on Acsl1 gapmer-derived hepatotoxicity. Acsl1 gapmer (10 mg/kg) was subcutaneously administrated 2 days after injection of siRNA-invivofectamine complex, and the liver and plasma were isolated on day 10 after ASO administration. Plasma ALT level (**a**); plasma AST level (**b**); expression level of *RnaseH1* mRNA (**c**); Western blot analysis of RnaseH1 and Acsl1 protein (**d**). Two homogenates of liver in each group were analysed using β-actin as internal standard. Expression level of *RnaseH1* mRNA (**e**) and *RnaseH2a* mRNA (**f**). *In situ* detection of apoptosis (**g**). Liver sections of mice that received both siRNA and Acsl1 gapmer was subjected to TUNEL assay and observed under confocal laser microscopy (TM; transmission, Scale bar: 5 μm). (n = 4, mean + S.D., *p < 0.05, Mann–Whitney *U* test, vs si-N.C. + gapmer).

The impact of complete suppression of the ALT/AST increase by RNaseH1 knockdown led us to test another hepatotoxic ASO, the Acsl1 gapmer. Mice were administered siRNA–invivofectamine 2 days before, and sacrificed 10 days after, gapmer injection. Similar to the findings for the GR gapmer, plasma ALT/AST levels were dramatically reduced in si-RNaseH1-administered mice (Fig. 3a,b). *Acsl1* expression was suppressed in every Acsl1 gapmer-administered group, but the knockdown effect was about 20% lower in the si-RNaseH1-injected group than in the other siRNA-treated groups (Fig. 3c). Western blot analysis showed that Acsl1 protein expression was effectively reduced by gapmer administration. The reduction in protein levels was a little weaker in si-RNaseH1-treated mice than in si-N.C.- or si-RNaseH2a-treated mice (Fig. 3d). qRT-PCR showed that the expression of *Rnaseh1* and *Rnaseh2a* mRNA in each siRNA-treated mouse liver was approximately 50% of that in controls (Fig. 3e,f). Saline administration after siRNA treatment did not cause any significant changes. Although we also examined RNaseH2, the silencing of RNaseH2a exhibited no significant effects on either gapmer hepatotoxicity or knockdown activity. LNA-modified gapmers are known to cause apoptosis in mouse liver^7^. To further investigate gapmer-mediated hepatotoxicity, we performed the TUNEL assay on liver cryosections prepared from mice that received injections of siRNA and Acsl1 gapmer. A positive signal was observed occasionally (1 in 30–40 visual fields) in si-N.C.- or si-RNaseH2a-treated gapmer-injected mice (Fig. 3g). However, no signal was observed in si-RNAseH1-treated sections.

**Figure 4:**
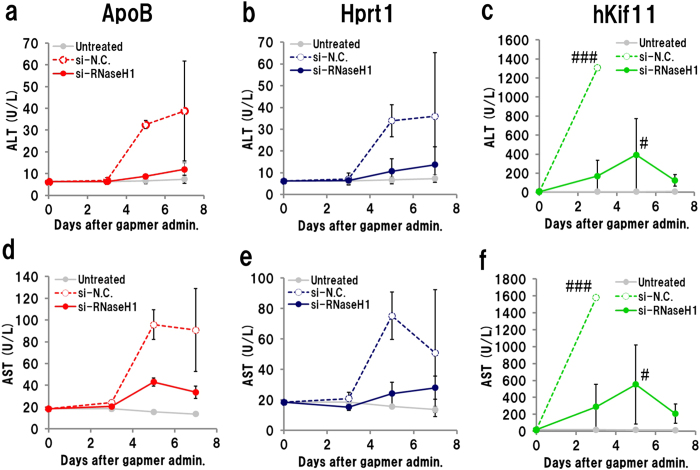
Effect of RNaseH1 knockdown on other hepatotoxic gapmers. Mice that had received siRNA–invivofectamine complex were administrated hepatotoxic gapmers targeting ApoB (20 mg/kg), Hprt1 (80 mg/kg), or human Kif11 (80 mg/kg). Upper panels show plasma ALT; lower panels show plasma AST. ApoB (**a**,**d**), Hprt1 (**b**,**e**), and human Kif11 (**c**,**f**). (n = 3, mean ± S.D.) ^#^Mouse was euthanized because of acute weight loss.

To better understand the effect of RNaseH1 knockdown on hepatotoxicity, we repeated the RNaseH1-knockdown experiment using other toxic gapmers, targeting apolipoprotein B (ApoB; ref. 37), hypoxanthine phosphoribosyltransferase 1 (Hprt1), and human kinesin family member 11 (human Kif11; ref. 38). Two days after injection of the si-RNaseH1-invivofectamine complex, gapmers were administered subcutaneously, and plasma ALT/AST levels were monitored. Administration of si-RNaseH1 significantly suppressed the increase in plasma ALT/AST levels caused by all three gapmers (Fig. 4a–f). These results suggest that the hepatotoxicity of gapmers could be derived from on- and/or off-target knockdown.

### Knockdown of Acsl1 mRNA by GalNAc3-conjugate siRNA

**Figure 5:**
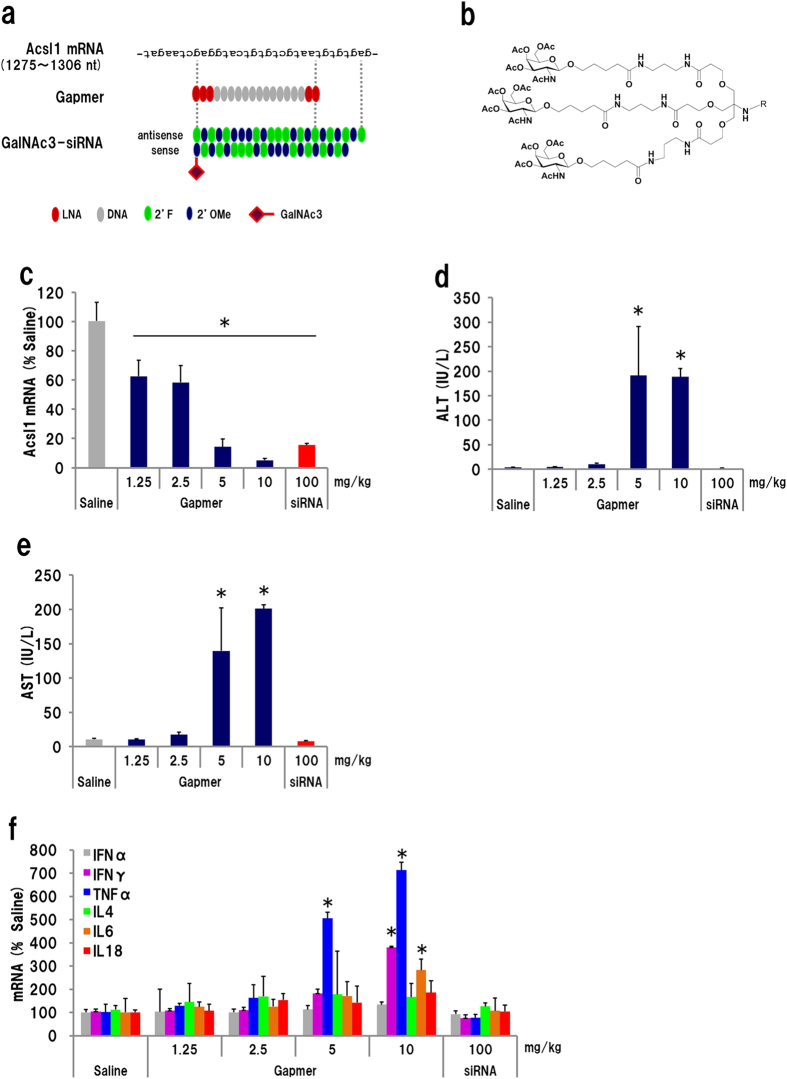
Comparison of hepatotoxicity and knockdown activity using a gapmer with GalNAc3-siRNA. (**a**) Schematic of GalNAc3-siRNA. The siRNA was designed to target almost same position of *Acsl1* mRNA as does the gapmer. To avoid degradation, 2′-fluoro- (2′F) and 2′-O-methyl (2′OMe) nucleotides were utilized. (**b**) Structure of GalNAc3 probe. (**c**) *Acls1* mRNA level. Administration of GalNAc3-siRNA resulted in almost the same level of knockdown as 5 mg/kg/day of gapmer. (**d**) Plasma ALT and (**e**) AST levels. (**f**) Expression profile of cytokines. (n = 3, mean + S.D., *p < 0.01, Mann–Whitney *U* test, vs saline).

Triantennary *N*-acetyl galactosamine (GalNAc3)-conjugated, fully chemically modified siRNA is known to effectively reduce target RNA expression in liver^39^,^40^. We compared both the knockdown activity and hepatotoxicity of GalNAc3-siRNA and the gapmer targeting the same position of the Acsl1 mRNA (Fig. 5a,b). GalNAc3-siRNA administration achieved almost the same knockdown effect as did 5 mg/kg/day (15 mg/kg in total) of gapmer treatment (approximately 85% mRNA reduction); siRNA treatment did not result in any increase in plasma ALT/AST levels, while gapmer injection resulted in robust hepatotoxicity (Fig. 5c–e). We examined the mRNA level of inflammatory cytokines IFNα, IFNγ, TNFα, IL4, IL6, and IL18 by qRT-PCR. The expression of IFNγ, TNFα, and IL6 was significantly enhanced by gapmer (≥5 mg/kg/day) but not by siRNA (Fig. 5f). We also measured plasma IFNα and IFNγ protein using enzyme-linked immunosorbent assay kits, but they were at background level. This suggests that the cause of the hepatotoxicity could depend on the gapmer’s unique knockdown mechanisms.

### Off-target knockdown by the Acsl1 gapmer

Gapmers are considered to digest target RNA inside the nucleus, because RNase H1 localizes in nuclei^41^. To confirm that the gapmer cleaves intra-nuclear RNA, we quantified the introns of Acsl1 pre-mRNA by qRT-PCR on total RNA from the liver of mice that received gapmer or GalNAc3-siRNA administration. Primers were designed to amplify portions of introns 1, 2, 3, 4, 7, 10, 12, and exons 1 and 12 (Supplementary Table S4).The expression level of the introns of *Acsl1* pre-mRNA was reduced to approximately 40% of the saline control level at every site, indicating that the gapmer digests *Acsl1* pre-mRNA inside the nucleus. Administration of GalNAc3-siRNA did not change the expression level of introns, presumably because Ago2, an endogenous nuclease that functions with siRNAs, localizes only in the cytoplasm (Supplementary Fig. S2a,b).

**Figure 6:**
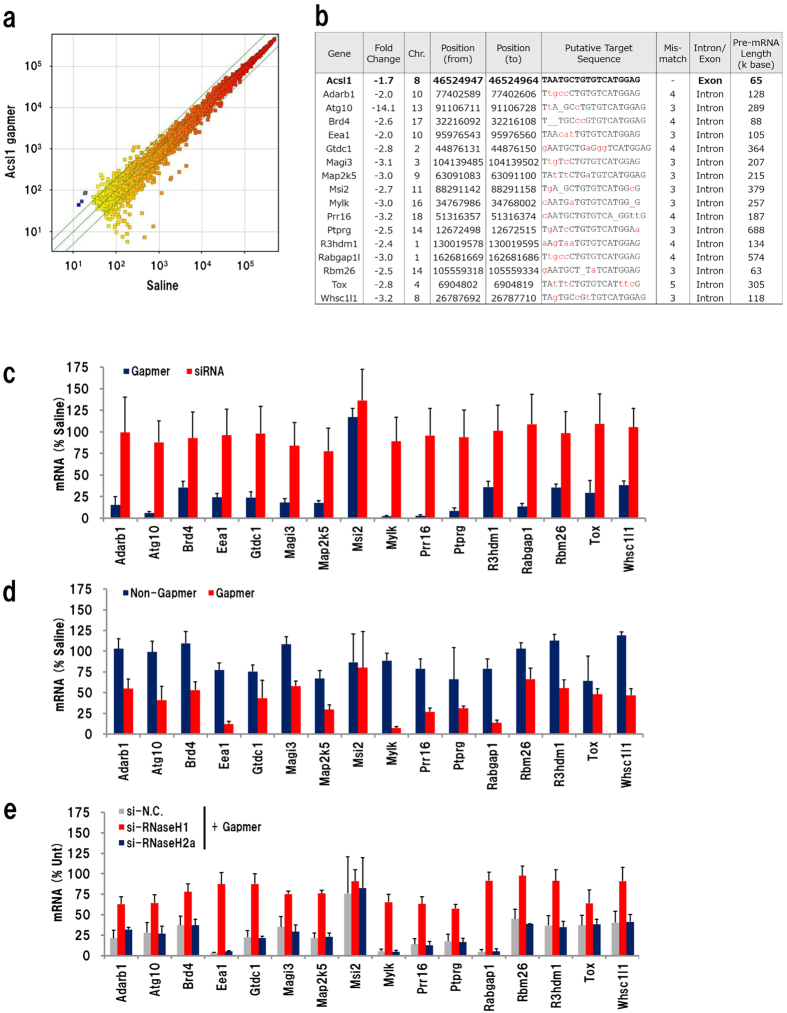
Analysis of off-target knockdown. (**a**) Scatter plot of expression level data from Acsl1 gapmer- and saline-administrated mice liver. (**b**) Presumptive off-target protein-coding genes of the Acsl1 gapmer which showed greater than two-fold significant change. qRT-PCR analysis of the gene expression in liver of gapmer- or GalNAc3-siRNA-treated mice (**c**), non-gapmer- or gapmer-treated mice (d), and si-RNaseH + gapmer (**e**) (n = 3 or 4, mean + S.D.).

Next, we investigated the off-target knockdown effect of the Acsl1 gapmer. We performed a microarray analysis using liver RNA isolated 24 h after injection of 20 mg/kg Acsl1 gapmer. To avoid the fluctuation in gene expression caused by the hepatotoxic effect, we collected liver samples at 24 h after injection because hepatotoxicity of gapmers can be observed at least 3–4 days after administration (*see* Figs 1 and 4). Plasma ALT and AST levels did not increased at this time point. A scatter plot of the microarray analysis indicated that the expression of few genes was changed by the Acsl1 gapmer; only 16 probes for protein-coding gene showed a greater than two-fold significant decrease, while no probe showed significant increase (Fig. 6a). The transcriptome analysis did not show upregulation of immune-related genes. Decrease of Acsl1 mRNA was less than two-fold (1.7 fold). All of those genes had similar sequence (containing 3–5 mismatches) to Acsl1 gapmer-targeting site in intron of the pre-mRNA, and these pre-mRNAs were considerable long, most of them were over 100,000 base (Fig. 6b). To examine off-target knockdown of those genes, we performed qRT-PCR of total RNA obtained from mice that received gapmer and GalNAc3-siRNA. Interestingly, almost all genes showed robust decrease in mRNA expression in gapmer-treated mice, while no significant change was observed in GalNAc3-siRNA-injected mice (Fig. 6c). To gain further understanding of the off-target knockdown, we investigated these genes in mice that received administration of the other oligonucleotides. The mice received ‘non-gapmer’ did not show any significant knockdown, while gapmer reduced most of these gene expressions (Fig. 6d). We also examined the effect of si-RNAseH1-invivofectamine treatment, and found that the silencing of si-RNaseH1 significantly suppressed the off-target effects caused by the gapmer (Fig. 6e). These results suggested that the gapmer recognizes and digests pre-mRNAs that possess a target-like sequence, and this off-target knockdown would be the main cause of hepatotoxicity.

Additionally, using an online mouse genome database, we identified 395 genomic positions that were similar to the Acsl1 gapmer-targeting sequence which contains 2 or fewer mismatches. Of these, 185 were located in RNA-encoding regions, and of these, 155 were in protein-encoding genes (9 were in exons including Acsl1, 146 were in introns; Supplementary Table S5). Using microarray data, we investigated the expression of the 155 genes that possess gapmer target-like sequences, and found that 9 of them were genes likely to be knocked down (Supplementary Fig. S4a). To confirm off-target knockdown of those genes, we performed qRT-PCR of total RNA obtained from mice that received 5 mg/kg/day gapmer and found that seven genes showed a significant decrease in mRNA expression (Supplementary Fig. S4b). There was no significant change in these 9 genes in GalNAc3-siRNA-injected mice. Next we investigated the total RNA of non-gapmer administrated mice, and found that none of these 9 genes were knocked down (Supplementary Fig. S4c). Silencing of hepatic RNaseH1 using si-RNAseH1-invivofectamine complex strongly suppressed the off-target knockdown caused by the gapmer (Supplementary Fig. S4d).

## Discussion

Many nucleotide- and nucleoside-analogues have been developed to treat viral infections and cancer, but these compounds cause cellar toxicity related to the inhibition of essential molecules such as host enzymes or transporters^42^. It is possible that the metabolites of LNA gapmers—monomeric or polymeric LNA with PS bonds—might inhibit essential subcellular events. In fact, one publication has suggested that the hepatotoxicity of LNA gapmers might be because of both PS and LNA modification at the 3′ end of the gapmer^7^. However, our experiments indicated that the GR and Acsl1 gapmers lost their hepatotoxicity and knockdown activity simultaneously upon substituting two deoxyribonucleotides at the centre of the gap region for LNA. This means that the chemical characteristics of the GR and Acsl1 gapmers, such as PS and LNA modification, as well as their metabolites, would not induce biochemical/biophysical toxicity

The innate immune response is a TLR-dependent immunological reaction caused by certain oligonucleotide sequences^43^. Oligonucleotides harbouring an unmodified CG dinucleotide (CpG motif), as well as some non-CpG-containing motifs, are recognized by TLR9, activate inflammatory reactions, and lead to an increase in plasma ALT/AST^44^,^45^. Additionally, some oligonucleotides are TLR9-independent, presumably involving other TLR-dependent immune reactions. Gapmers also induce an innate immune response; chemical modification, as well as substitution of nucleotides at an immune-reactive sequence of the gapmer, strongly suppresses TLR-dependent immune responses^46^,^47^,^48^. However, the hepatotoxicity caused by the gapmers used in this study was probably not caused by sequence-dependent immune responses, because the hepatotoxicity of all the gapmers was significantly suppressed by the knockdown of RNaseH1.

We used five gapmers in this study; GR, Acsl1, ApoB, Hprt1, and human Kif11. The knockdown of GR using MOE-modified gapmers shows therapeutic effects for treatment of diabetes without showing any toxic side effects^49^,^50^. Liver-specific knockout of *Acsl1* gene decreases triacylglycerol synthesis and β–oxidation without hepatotoxicity^51^. GalNAc3-Acsl1-siRNA achieved the same level of knockdown as did the gapmer, and without toxicity. Administration of the ApoB gapmer (1–2 mg/kg/week) reduces non high density lipoprotein cholesterol without increasing serum liver toxicity markers, accompanied by potent knockdown of *ApoB* mRNA^37^. *Hprt1* is non-essential housekeeping gene; its gene locus is commonly used for production of knock-in mouse. The human Kif11 gapmer used here does not have target sequence in mouse. Therefore the toxicity of those gapmers cannot originate from on-target knockdown activity.

A recent publication by Burel *et al.* suggests that the hepatotoxicity of gapmers is mediated by RNase H1-dependent promiscuous reduction of RNAs^27^. They demonstrated that long pre-mRNA transcripts tended to be degraded by off-target effects. In fact, both intronic and exonic off-target RNA cleavage have been observed in cultured cells^52^. It has been demonstrated that non-target RNA containing even three mismatches could be degraded by gymnastic transfection of gapmers into cultured cells. In this study, we analysed in detail off-target knockdown using the Acsl1 gapmer as a model toxic gapmer *in vivo*, and found that various mismatch-containing pre-mRNAs were knocked down in mouse liver. There were no common genes which were downregulated by toxic gapmers in previous report by Burel *et al.*^27^. The maximum number of mismatch was 5 (*Tox* mRNA), indicating that the target recognition of LNA gapmer would not be highly specific. It implies that more RNAs—those containing 6 or more mismatches—might also be digested by RNaseH1. In line with recent findings, the data shown here offer deep insights that gapmers cleave mismatch-containing pre-mRNA *in vivo*; this is considered to be a fundamental cause of the hepatotoxicity. Additionally, comparative analyses between GalNAc3-siRNA and gapmers imply that siRNAs might be safer than gapmers for the knockdown of cytosolic mRNAs.

There are two approaches that could help avoid off-target knockdown. One is in silico analysis to predict putative off-target cleavage^53^. This may enable us to screen out the gapmers that would be likely to cleave a large number of non-target RNAs. However, because gapmers are short (13–20 nucleotides), there would be many putative targets when considering three or more mismatches. The accumulation of data on off-target knockdown by various gapmers would facilitate fine-tuning of a database for computational analysis. The second approach is using chemical biology. Thiothymidines, for instance, are known to enhance thermodynamic stability of T–A base pairing, but not a naturally occurring T–G mismatch^54^. A novel nucleoside analogue, which dramatically improves target sequence-recognition, could reduce the off-target effects of gapmers. The further development of both *in silico* analysis and chemical biology will open up new possibilities for using gapmer ASOs as safe and effective therapeutics.

## Methods

### Mouse

C57BL6/j male mice (5-week old, CLEA Japan, Tokyo, Japan) were purchased and maintained according to the instruction of Association for Assessment and Accreditation of Laboratory Animal Care International. All experimental protocol were approved by the Institutional Animal Care and Use Committee of Shionogi Co., Ltd., and all experiments were performed in accordance with the Committee’s guidelines. Anaesthesia was induced by intraperitoneal administration of approximately 20 μg of sodium pentobarbital, and then the liver was isolated. For western blot analysis, approximately 100 mg liver was immediately frozen in liquid nitrogen and stored at −80 °C. Approximately 40 mg of liver sample was stored in RNA Later (Qiagen, Hilden, Germany) at 4 °C for overnight and then at −80 °C for quantitative reverse-transcription PCR (qRT-PCR) and microarray analysis. The rest was fixed in 4% paraformaldehyde and embedded in Optimal Cutting Temperature compound (Sakura Finetek, Tokyo, Japan) for histological observation.

### ASOs and siRNA

LNA-modified ASOs were supplied from Gene Design (Osaka, Japan). Detailed sequence information is shown in Supplementary Table S1. Lyophilized ASO was dissolved in saline. ASOs were administrated subcutaneously into the back of 5-week-old mice at indicated doze. RNaseH1, H2a, and negative control siRNA (si-RNaseH1, si-RNaseH2a, and si-N.C., respectively) were purchased from Life Technologies (Carlsbad, CA). The siRNAs were formulated with invivofectamine 2.0 (Life Technologies). siRNA complexation solution was prepared by mixing 250 μl of 3 mg/ml siRNA with 250 μL of complexation buffer, then 500 μl of invivofectamine 2.0 reagent was added. The mixtures were incubated for 30 min at 50 °C, and dialysed against 1000× volume of phosphate-buffered saline (PBS) using Float-A-Lyzer G2 (Spectrum Laboratories, Rancho Dominguez, CA) for 1 h. The siRNA–invivofectamine complex, with a final concentration of 7 mg siRNA/kg, was injected through tail vein at 2 days prior to *s*.*c*. injection of ASOs. We designed a fully chemically modified, triantennary *N*-acetylgalactosamine (GalNAc3)-conjugated siRNA, which targets the same position of the target mRNA as does the gapmer (Supplementary Table S2). Both chemically modified siRNA and GalNAc3 probe were synthesized as previously described^39^,^54^. GalNAc3-siRNA (without any formulation such as invivofectamine) was injected subcutaneously every 3 days, and mice were sacrificed at 4 days after final administration.

### Measurement of plasma ALT/AST

Mouse plasma was collected from the tail vein routinely, and from the vena cava at the end point. ALT and AST were measured using Transaminase CII-test Wako (Wako Pure Chemical Industries, Osaka, Japan), according to the manufacture’s instruction for 96-well format. Plasma ALT and AST levels are shown in international unit.

### RNA Purification and qRT-PCR

Total RNA was purified from the RNAlater-treated frozen liver samples using an RNeasy 96 Universal kit (Qiagen) according to the manufacturer’s instructions. In brief, liver tissue was homogenized in 750 μl Qiazol reagent. The homogenate was mixed with 150 μl chloroform and the supernatant, containing the RNA, was applied to an RNeasy 96 well plate. cDNA was synthesized from 1 μg total RNA using a Superscript III First Strand Synthesis kit (Life Technologies). qRT-PCR was conducted using SYBR Premix Ex Taq II (Takara Bio, Shiga, Japan) according to the manufacturer’s instructions on an ABI7900HT Real Time PCR system (Applied Biosystems, Foster City, CA). Quantified mRNA levels were normalized to *Gapdh* and are presented relative to a saline or untreated control. Primers for conventional qRT-PCR are listed in Supplementary Table S3.

### Western Blotting

Liver tissues were homogenized in a 5× volume of radio-immunoprecipitation assay buffer (Sigma-Aldrich, St. Louis, MO) using a Tissue Lyzer with 7-mm stainless steel beads (Qiagen). The protein concentration of the homogenate was measured using a BCA Assay kit (Thermo Fisher Scientific, Waltham, MA). Homogenate containing 12.5 μg protein was subjected to SDS-PAGE, electrically transferred to polyvinylidene difluoride membrane, and immunoblotted using primary antibodies for RNaseH1 (Proteintech, Rosemont, IL; dilution factor 1:1200), Acsl1 (Cell Signaling Technology, Beverly, MA; dilution factor 1:1000), or β-actin (Sigma-Aldrich; dilution factor 1:1000), followed by horseradish peroxidase-conjugated secondary antibodies (GE Healthcare, Little Chalfont, UK). The blots were visualized utilizing chemiluminescence with ECL Prime (GE Healthcare).

### Terminal deoxynucleotidyl transferase dUTP nick end labeling (TUNEL) Assay

Apoptotic cells in the liver tissue were detected by TUNEL assay, using an *In Situ* Cell Death Detection kit, Fluorescein (GE Healthcare), according to the manufacture’s instruction. In brief, 10 μm-thick cryosections were prepared from mouse liver, then the sections were incubated in 0.1% citric acid containing 0.1% Triton-X 100 on ice for 2 min, washed twice in PBS, and then immersed in TUNEL reaction solution at 37 °C for 60 min. After washing three times in PBS, sections were observed under confocal microscope FV500 (Olympus, Tokyo, Japan).

### Microarray analysis and database screening

Total RNA isolated from mouse liver was analysed using a whole mouse genome (4 × 44 K) oligo microarray kit (Agilent Technology, Santa Clara, CA) according to the manufacturer’s instructions. RNA transcripts containing a sequence similar to the target sequence of the Acsl1 gapmer (two or fewer mismatches) were screened using the ultrafast sequence searching database gggenome (http://gggenome.dbcls.jp/en/).

### Statistical Analysis

The data of experiments were assessed with the Mann–Whitney *U* test. Asterisks in figures indicate significant difference from the control group.

## Additional Information

**How to cite this article**: Kasuya, T. *et al.* Ribonuclease H1-dependent hepatotoxicity caused by locked nucleic acid-modified gapmer antisense oligonucleotides. *Sci. Rep.* **6**, 30377; doi: 10.1038/srep30377 (2016).

## Supplementary Material

Supplementary Information
