# Supplementary material for: Ribonuclease H1-dependent hepatotoxicity caused by locked nucleic acid-modified gapmer antisense oligonucleotides
Source: Sci Rep. 2016 Jul 27;6:30377. doi: 10.1038/srep30377 (PMC4961955; doi:10.1038/srep30377)
Supplement: Supplementary Information [file srep30377-s1.doc]

**Supplementary Information**

**Ribonuclease H1-dependent hepatotoxicity caused by locked nucleic acid-modified gapmer antisense oligonucleotides**

Takeshi Kasuya^1^, Shinichiro Hori^1^, Ayahisa Watanabe^2^, Mado Nakajima^1^, Yoshinari Gahara^1^, Masatomo Rokushima^1^, Toru Yanagimoto^1^ and Akira Kugimiya^1^

^1^Discovery Research Laboratories for Innovative Frontier Medicines,

^2^Research Laboratory for Development, Shionogi & Co., LTd., Osaka, 561-0825, JAPAN

**Supplementary Figure S1.**


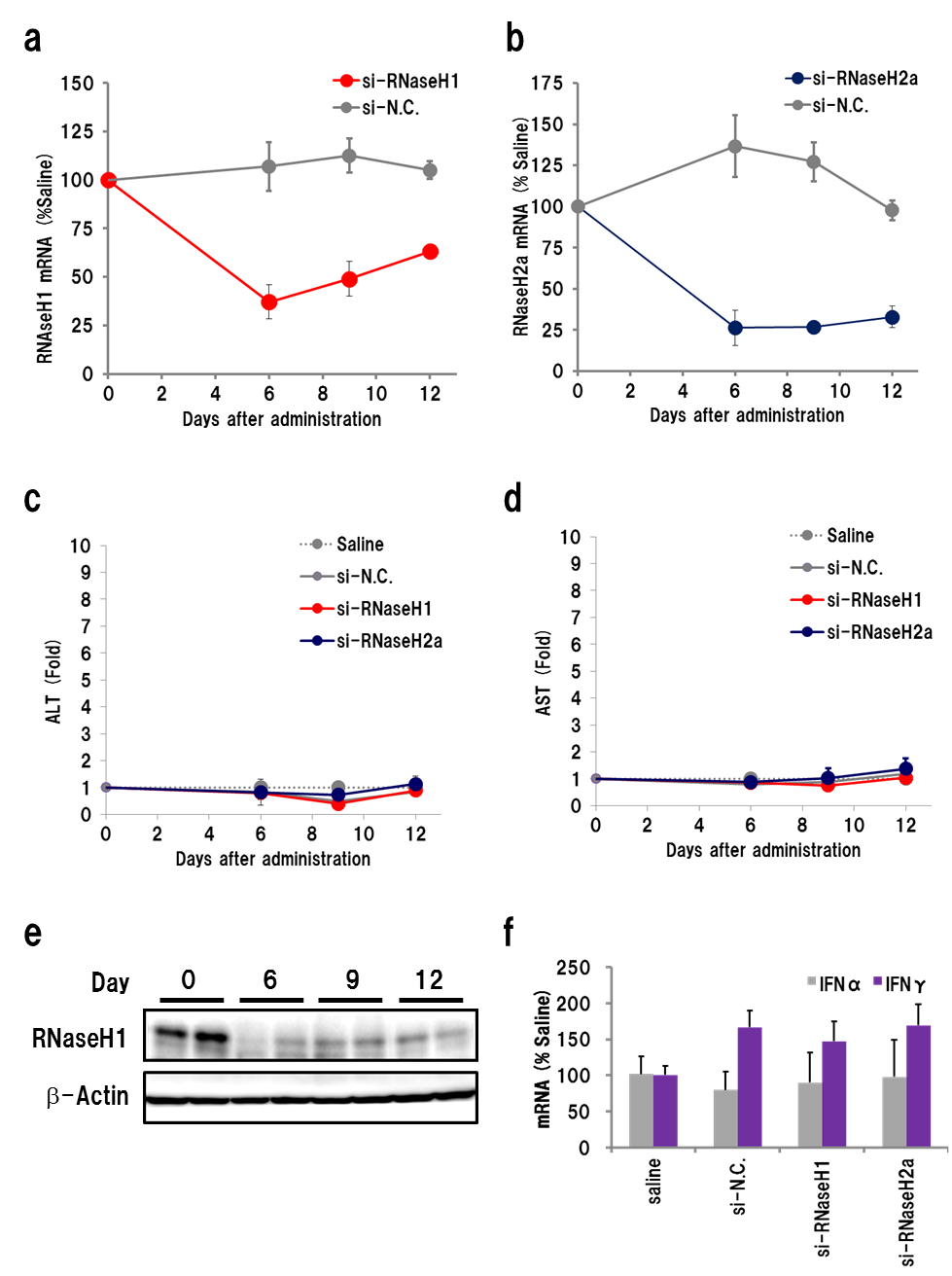
*In vivo* knockdown of RNaseH1 and RNaseH2a in mouse liver. Mice were sacrificed on 6, 9 and 12 day after administration of siRNA-invivofectamine complex. Stealth RNAi™ siRNA was used for the experiment. Expression level of *RnaseH1* (a) and *RnaseH2a* mRNA (b); plasma ALT level (c); plasma AST level (d); Expression level of RNaseH1 protein (e); *IFNα* and *γ* mRNA (F) (n=3, mean±S.D.)

**Supplementary Figure S2.**

Cytotoxicity of si-RNAseH1 and H2a *in vitro*. Mouse liver-derived Hepa1c1c7 cells were transfected 5 nM of si-N.C., RNaseH1, and H2a using Lipofectamine 3000 (Invitrogen). After 48 of transfection, cell viability (a) and knockdown effect (b) were measured using Cell Counting kit-8 (Dojindo, Tokyo, Japan) and qRT-PCR, respectively (n = 5, mean + S.D.).


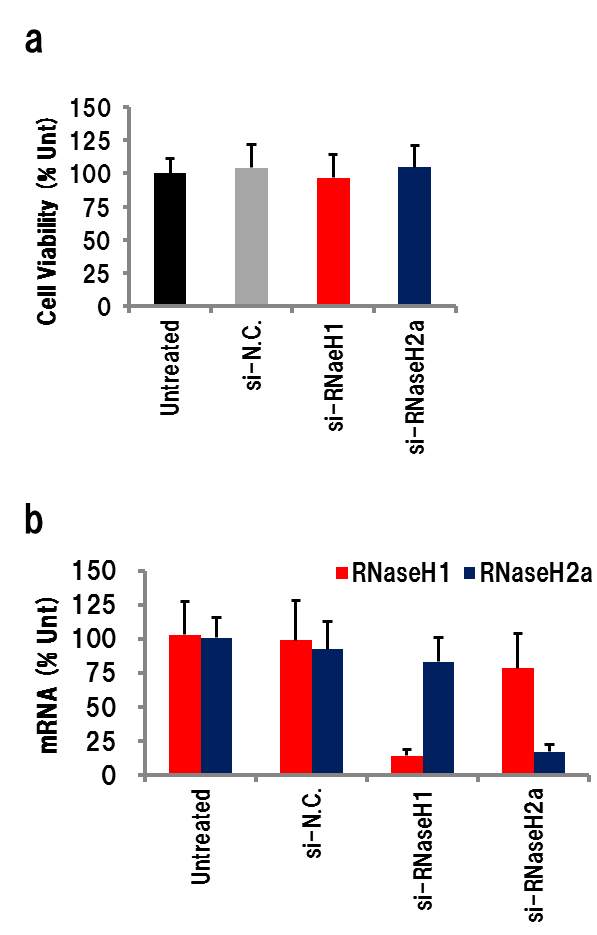


**Supplementary Figure S3.**

Quantitation of the introns of *Acsl1* pre-mRNA. The liver total RNA from gapmer (a) or GalNAc3-siRNA (b) recipient mice were analysed by qRT-PCR using 14 intronic and 2 exonic primer pairs. *Acsl1* pre-mRNA is schematically illustrated in each figure (Line; intron, Square; exon). Both all introns and exons were decreased in gapmer-treated mice, whereas only exons were decreased in siRNA-treated ones (n=3, mean +/- SD). **
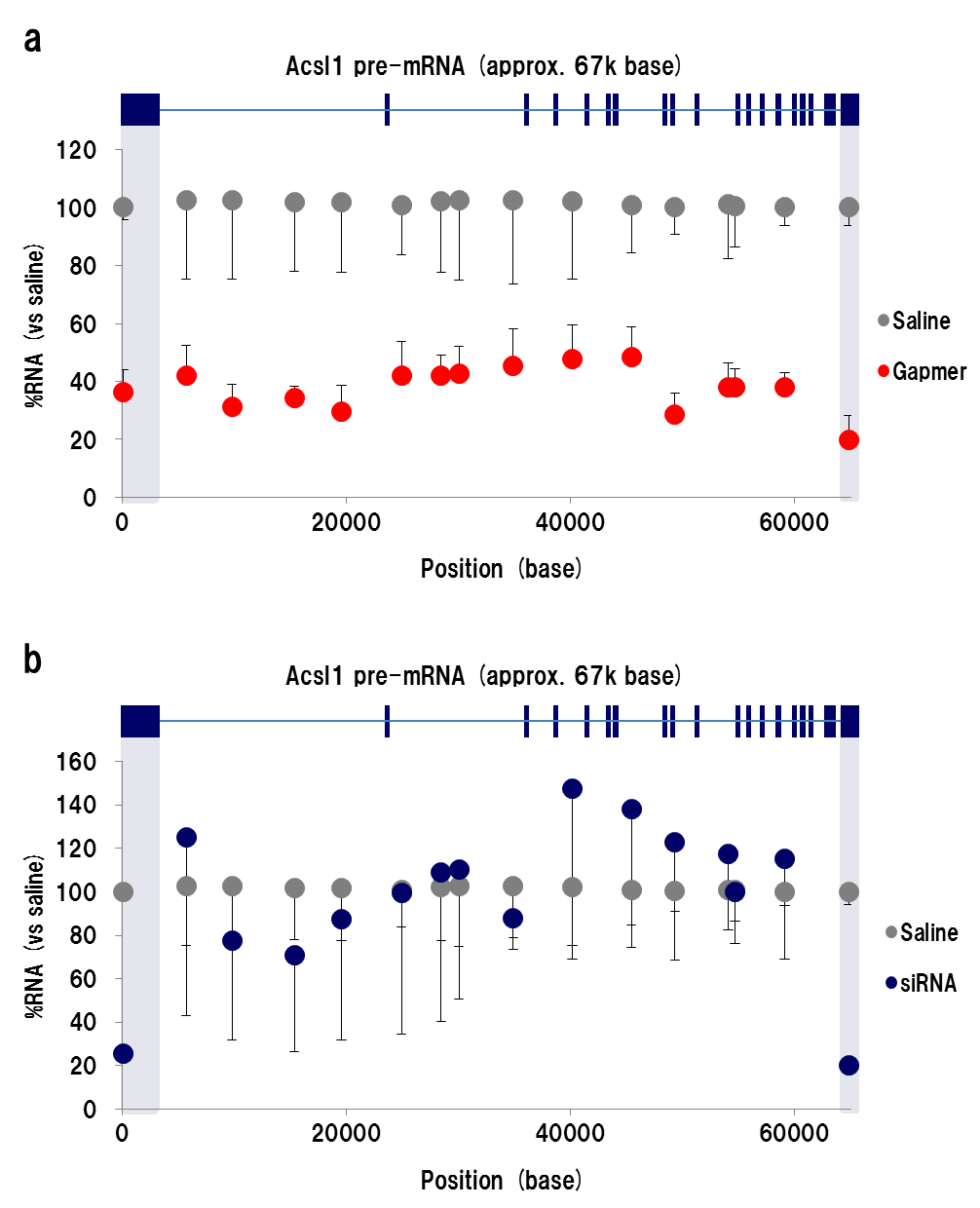
**

**Supplementary Figure S4.**

*Analysis of off-target knockdown*. (a) Presumptive off-target genes of the Acsl1 gapmer extracted from microarray data: those that shows similar fold-change to Acsl1. qRT-PCR analysis of the gene expression in mice treated with gapmer (b), non-gapmer or gapmer (c), and both si-RNAaseH and gapmer. (n=3 or 4, mean + S.D.).

a


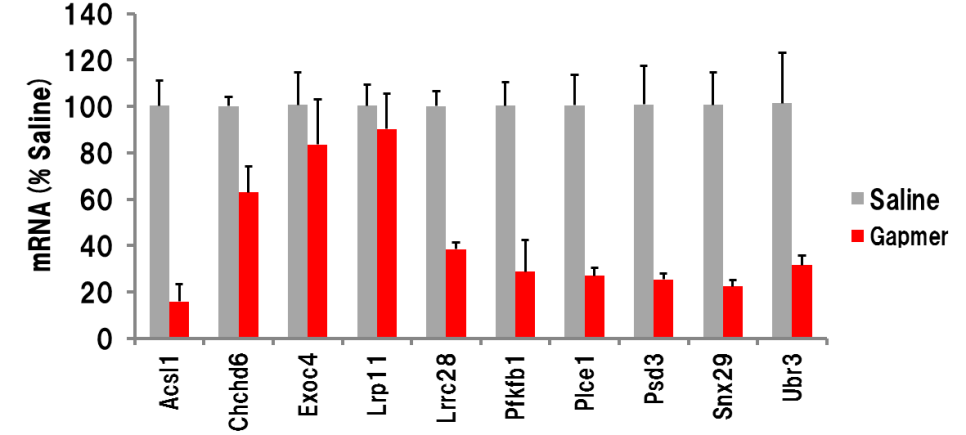

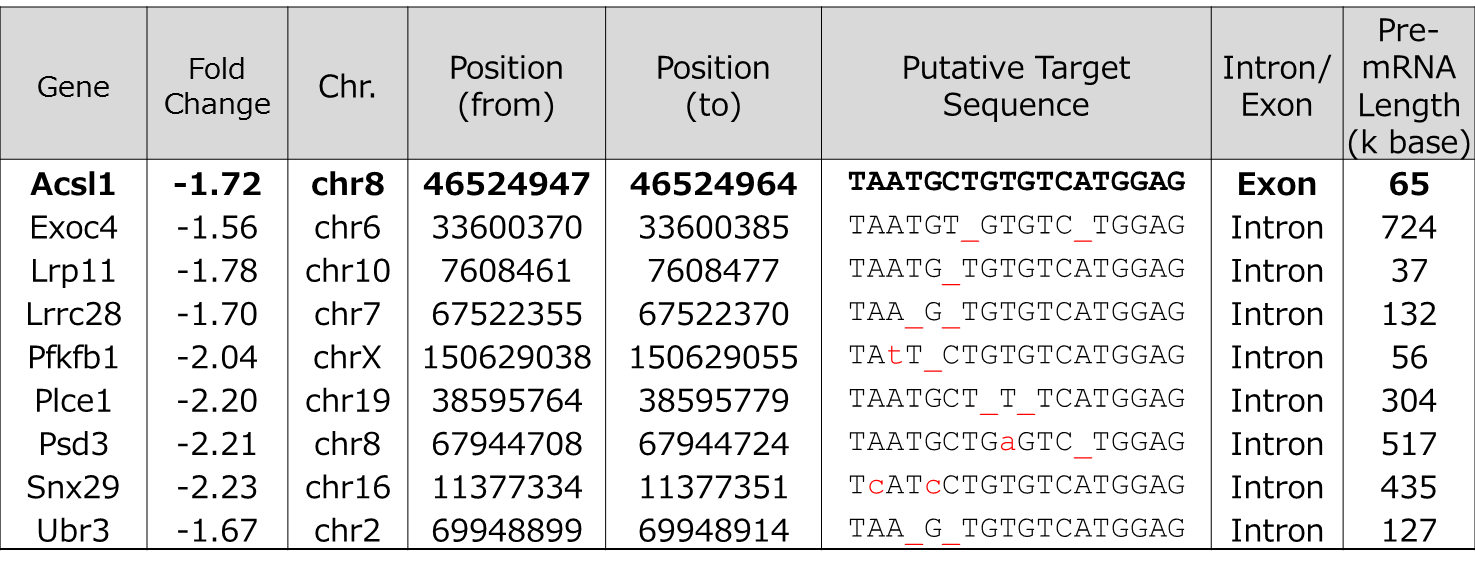


b

c

d

+ Gapmer


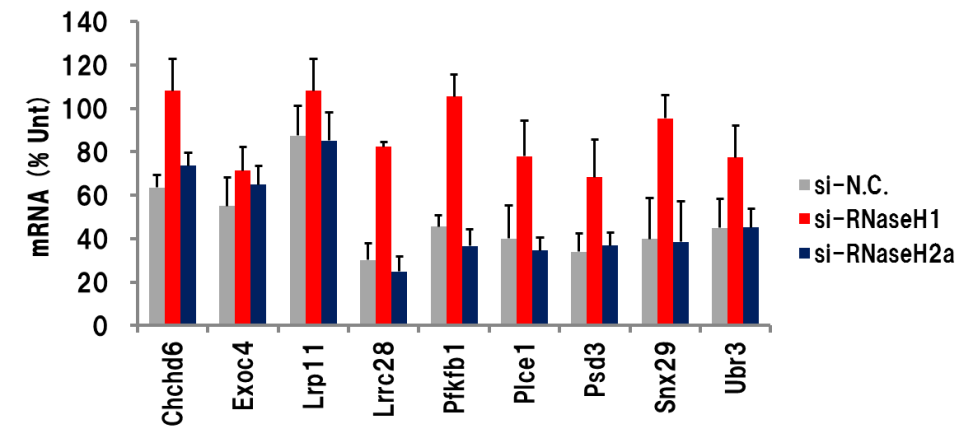

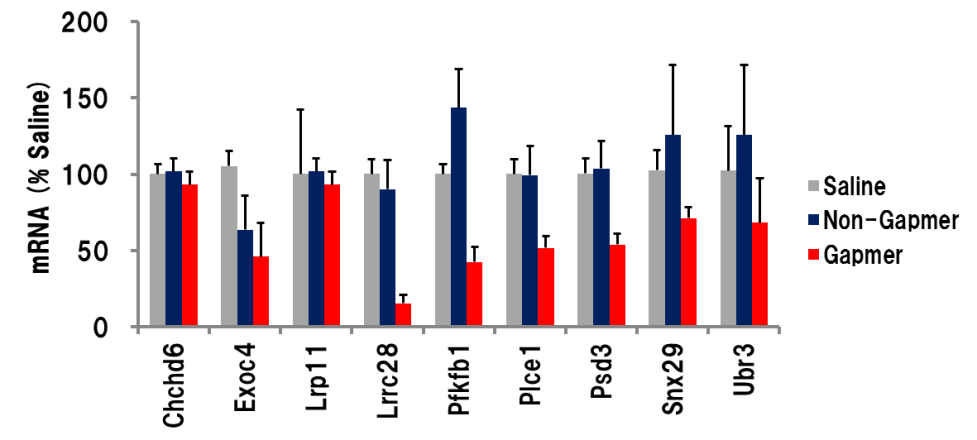


**Supplementary Table S1.**

The sequence of ASO used in this study. All of oligonucleotides were fully modified with phosphorothioate. *Human Kif11 gapmer is 2-mer shorter than original 2’MOE gapmer (ISIS183881). Upper case; LNA. Lower case: DNA.

| **Target** | **Type of Oligonucleotide** | **Sequence** | **Target RNA**  **(GeneBank Accession No.)** | **Target posision** | **Ref.** |
| --- | --- | --- | --- | --- | --- |
| Acsl1 | Gapmer  Non-Gapmer | CTCcatgacacagcaTTa  CTCcatgaCAcagcaTTa | NM_007981 | 1222-1239 | - |
| Glucocorticoid Receptor | Gapmer  Non-Gapmer | GTCtctttaccTGG  GTCtctTTaccTGG | NM_008173 | 677-690 | 17 |
| ApoB | Gapmer | GCattggtatTCA | NM_009693 | 10136-10148 | 37 |
| Hprt1 | Gapmer | GGtcataaccTGG | NM_013556.2 | 189-201 | - |
| *human Kif11 | Gapmer | TCCaagtgctactgtAGT | NM_004523.3 | 2052-2069 | 38 |

**Supplementary Table S2.**

Sequence and chemical modification of GalNAc3-siRNA. GalNAc3-probe was conjugated at 3’end of sense strand *via* phosphorothioate bond. Upper case; 2’OMe, Lower case; 2’F, ^; phosphorothioate.

| **Target** | **Type of Oligonucleotide** | **Sequence** | **Target RNA ID** | **Target posision** |
| --- | --- | --- | --- | --- |
| Acsl1 | sense  antisense | GuGuAaUgCUGuGucaUgGaG cUcCaUGAcAcagCaUuAcAc^A^c | NM_007981 | 1217-1239 |

**Supplementary Table S3.**

The list of primers used in this study for conventional qRT-PCR.

| **Gene** |  | **Primer Sequence** |
| --- | --- | --- |
| Acsl1 | Forward  Reverse | GCAGCGGCATCATCAGAAAC  TGTCACCATCAGCCGGACTC |
| Adarb1 | Forward  Reverse | GAGGTCTCCGCCAGTCAAGAA  TCAGTGCTGCTGGAACCTGTG |
| Atg10 | Forward  Reverse | CTTGTAGTTACCAAGTGCCGGTTC  AATGGTTGCCCAAGTATTGGATG |
| Brd4 | Forward  Reverse | TGCACGACTACTGTGACATCATCAA  GCACCAAATTCCTGGGCATC |
| Chchd6 | Forward  Reverse | TGCATGAAGTGCTTCTGTGCTC  GGCCATGGTCTGTGGTCCTA |
| Eea1 | Forward  Reverse | GAATCACTTGGACACAGCTCAACA  AGCTGATCCAACTGCACACTGAC |
| Exoc4 | Forward  Reverse | CAATACTTCCGGCGCATCAG  TGGTCCACCACCAGGTTCAG |
| Gapdh | Forward  Reverse | TGTGTCCGTCGTGGATCTGA  TTGCTGTTGAAGTCGCAGGAG |
| Glucocorticoid Receptor | Forward  Reverse | GTGAAATGGGCAAAGGCGATAC  CAGGGCAAATGCCATGAGAA |
| Gtdc1 | Forward  Reverse | GTTGTCATCTCAACAGCCAAGCA  TGGGTAGCAGCCACAGTACACAG |
| IFNα | Forward  Reverse | CTGTGCTTTCCTGATGGTCCTG  GGAATCCAAAGTCCTTCCTGTCCT |
| IFNγ | Forward  Reverse | CGGCACAGTCATTGAAAGCCTA  GTTGCTGATGGCCTGATTGTC |
| IL4 | Forward  Reverse | ACGGAGATGGATGTGCCAAAC  AGCACCTTGGAAGCCCTACAGA |
| IL6 | Forward  Reverse | CAACGATGATGCACTTGCAGA  CTCCAGGTAGCTATGGTACTCCAGA |
| IL18 | Forward  Reverse | TTCTGCAACCTCCAGCATCA  AGTGAAGTCGGCCAAAGTTGTCT |
| Lrp11 | Forward  Reverse | AGTCCAAGAGTGGCCAAGCAG  GTCGGCATGTCACCATGAGAA |
| Lrrc28 | Forward  Reverse | TAACCAGCTACAGTTCCTACCTCCA  GTCCACAGTGAGGTACTGCAGAGA |
| Magi3 | Forward  Reverse | TGGTGGAGATAGGCCTGATGAA  GACAACATCTGCATGGGTGTGA |
| Map2k5 | Forward  Reverse | CAGCTAATAGAGCCGCTGCAGATA  CACAGGGCTGGTGTGTTGAGA |
| Msi2 | Forward  Reverse | CTGGCAGACGAGGCTTTAGAGG  CGGCATTATTGGTGCTGAATTG |
| Mylk | Forward  Reverse | CCGTGGAGCTGACTGTGGAA  GCTTGGTGGCAAACTTTGGTG |
| Pfkfb1 | Forward  Reverse | ATGCCGACATGGTGAGAGTGA  AATGAAGTTGGCCAGGGCATAG |
| Plce1 | Forward  Reverse | CTGGGCATAAGCACCACCAAG  GTAGCCATCTCATCGTCCGTCA |
| Prr16 | Forward  Reverse | TTAGTCCTGGGCGACCTGAAG  GTCAGGGTGTCAATCTGGTCAAC |
| Psd3 | Forward  Reverse | GTTTAGCCGCCCACTTCTG  ACATCCTTGGCTTTGACCTTC |
| Ptprg | Forward  Reverse | TGCAGATCCGCAGACGAAAG  ACGCGAGCATGGTGGTCTAA |
| Rabgap1 | Forward  Reverse | GCCCAGGTCGAAACGTGAA  GAGCCAGAAATATTGGTGCGTTG |
| Rbm26 | Forward  Reverse | TGTGCCTGGGCATGCTGTA  AGCGTCATCAATCTGACAGTCCTC |
| RnaseH1 | Forward  Reverse | ACGAGCAGGAATTGGCGTTTAC  CTGAGCCTTGGCTTGCATGA |
| RnaseH2a | Forward  Reverse | CCCGAGACAAGGCTGTGAAGA  TGTGCTCCAACTGAACCGTACAA |
| R3hdm1 | Forward  Reverse | GCATGGACACATTCCACATCAA  CCACTCGAGTTATTCCGTCTGGTA |
| Snx29 | Forward  Reverse | AGAGCCTGGCCTTCGGTACA  ATGGCTTGGCGCAGTTCA |
| TNFα | Forward  Reverse | TATGGCCCAGACCCTCACA  GGAGTAGACAAGGTACAACCCATC |
| Tox | Forward  Reverse | CATGCAGGTCCAGACTGCCTTA  TGCACCCAGAACGCACGTA |
| Ubr3 | Forward  Reverse | AACCTGGAGGCTCTATGCAACAA  GTCTCGGCGGTAAACGGTTC |
| Whsc1l1 | Forward  Reverse | CAAGCAAGCCAGCAATCATTC  CATGAGCAATGCCAATGTCC |

**Supplementary Table S4**

The list of primers used for quantitation of the introns, as well as the first and final exons, for Acsl1 (pre-) mRNA.

| **Target Exon/Intron** | **Amplicon** |  | **Primer Sequence** |
| --- | --- | --- | --- |
| Exon1 | 99-171 | Forward  Reverse | CGGCCGCGACTCCTTAAATA  CACAGGCGGCTGTCACTG |
| Intron 1 | 5706-5839 | Forward  Reverse | CGTGCGACCATAGGGCTAAA  CAGCATTAGGGCAAGACCCA |
| Intron 1 | 9393-9523 | Forward  Reverse | GTCATCCCCAGGTGTCTTGG  AACAGAAGGTCATCGCCAGG |
| Intron 1 | 15351-15450 | Forward  Reverse | TGCAGCTCCCCTTAAGATCC  AACTTAGCCCTGGTCTTGGG |
| Intron 1 | 19566-19684 | Forward  Reverse | TGGTACACCCTAGGCACTCA  TAGCCGCCACAAGACAACTC |
| Intron 2 | 24913-25034 | Forward  Reverse | TCAGTGTTGGCTGGTTGTGT  CTTCCAGCTGAGTCCCAAGG |
| Intron 2 | 28465-28611 | Forward  Reverse | GTGGCTAGACCGAGTTCTTTC  CTCTACTGGCTAATTTCCTGTG |
| Intron 2 | 30052-30159 | Forward  Reverse | CCGTCCTTCTTGCCGTAAGT  TTGGATAGGCGGAAGTGCTC |
| Intron 3 | 34903-34981 | Forward  Reverse | TCCAGATGTTCTGTGTCGCC  AAGGCCAGCATACGTGACTC |
| Intron 4 | 40160-40333 | Forward  Reverse | CGGAAAGGACGCCAGATTGT  GTTCTGCCACCTGCAACAAA |
| Intron 7 | 45459-45614 | Forward  Reverse | TAAGGCTCTGAAGCCCTTGC  AGTCATGCACCACTATGCCC |
| Intron 12 | 54084-54190 | Forward  Reverse | TGTGAGTCCTGGTGCCAATC  CCACATCTATGGAGGCGGAC |
| Intron 12 | 54706-54802 | Forward  Reverse | CAGCATGAGCAGTTAGTCCAGC  GAGATGACAAAGAGTAGGTAAAGC |
| Intron 15 | 59143-59281 | Forward  Reverse | TGGAGGGGTCCTGAGTAGAC  CCACGCTCCCTCATAGGAAC |
| Exon 21 | 64881-65015 | Forward  Reverse | CGCTAATTTGTCACAACGGGG  TCCACTCACTCCAAATGCAGA |

**Supplementary Table S5.**

The list of RNA transcripts which contains similar sequence (including one or two mismatches) to the target sequence of Acsl1 gapmer.

| Chromosome | start | end | Sequence | Coding/ Noncoding | Gene name | Exon/ Intron |
| --- | --- | --- | --- | --- | --- | --- |
| **chr8** | **46524947** | **46524964** | **TAATGCTGTGTCATGGAG** | **C** | **Acsl1** | **Exon** |
| chr7 | 83775834 | 83775852 | TAATGTTGTGTtCATGGAG | C | 1700026D08Rik | Exon |
| chr13 | 77545167 | 77545182 | TAATGCT_T_TCATGGAG | C | 2210408I21Rik | Intron |
| chr9 | 41608483 | 41608499 | TA_TGCTTTGTCATGGAG | N | 2610203C20Rik | - |
| chr13 | 28480970 | 28480988 | TAATGCTGTGgTCATGtAG | N | 2610307P16Rik | - |
| chr2 | 85518690 | 85518706 | TGATGC_GTGTCATGGAG | C | 4833423E24Rik | Exon |
| chr16 | 25062417 | 25062433 | TAATGCT_TGTCATTGAG | N | A230028O05Rik | - |
| chr2 | 69284106 | 69284121 | TAATGCTGT_TCATG_AG | C | Abcb11 | Intron |
| chr2 | 48846165 | 48846181 | TAATGCTGTTTCATG_AG | C | Acvr2a | Intron |
| chr11 | 46105924 | 46105940 | TAAGGCTGTGTCA_GGAG | C | Adam19 | Intron |
| chr1 | 165550670 | 165550685 | TAATG_TGTGTCATGG_G | C | Adcy10 | Intron |
| chr7 | 76559516 | 76559532 | TA_TGCTGAGTCATGGAG | C | Agbl1 | Intron |
| chr4 | 110926438 | 110926455 | TTATCCTGTGTCATGGAG | C | Agbl4 | Intron |
| chr6 | 29903768 | 29903784 | TAATGCTGTGTGATGGAT | C | Ahcyl2 | Intron |
| chr15 | 27558630 | 27558645 | TAATG_TGTGTC_TGGAG | C | Ank | Intron |
| chr6 | 125768541 | 125768558 | TAATGCCGTGTCATAGAG | C | Ano2 | Intron |
| chr6 | 125940084 | 125940100 | AAATGCTG_GTCATGGAG | C | Ano2 | Intron |
| chr10 | 26755703 | 26755720 | TAATGCTGTGcTC_TGGAG | C | Arhgap18 | Intron |
| chr18 | 39343004 | 39343021 | TTATGCAGTGTCATGGAG | C | Arhgap26 | Intron |
| chr12 | 71095982 | 71095997 | TAA_GCTGT_TCATGGAG | C | Arid4a | Intron |
| chr2 | 106967921 | 106967938 | TAATGCTGGGTCCTGGAG | C | Arl14ep | Intron |
| chr11 | 81238255 | 81238271 | TAAT_CTGTGTCATTGAG | C | Asic2 (Bnc1) | Intron |
| chr11 | 81657507 | 81657522 | TAATGCTGTG_C_TGGAG | C | Asic2 (Bnc1) | Intron |
| chr4 | 66304198 | 66304214 | TAATGC_GTGTGATGGAG | C | Astn2 | Intron |
| chr18 | 64562824 | 64562839 | TAATGCTGT_TCATG_AG | C | Atp8b1 | Intron |
| chr4 | 32429941 | 32429956 | TAATGCTGTGTCA__GAG | C | Bach2 | Intron |
| chr11 | 120259020 | 120259037 | TAACGCTGTATCATGGAG | C | Bahcc1 | Intron |
| chr1 | 25263878 | 25263895 | TAATGCTTTCTCATGGAG | C | Bai3 | Intron |
| chr6 | 34481345 | 34481363 | TAATGCTGTGTCcATGGAG | C | Bpgm | Intron |
| chrX | 134576652 | 134576670 | TAAcTGCTATGTCATGGAG | C | Btk | Intron |
| chr2 | 14683640 | 14683657 | TAATGCTGCGTCATGGAG | C | Cacnb2 | Intron |
| chr14 | 12744604 | 12744619 | TAATG_TGTGTCA_GGAG | C | Cadps | Intron |
| chr6 | 23263177 | 23263193 | CAATGCTGTGTCATgGGAG | C | Cadps2 | Exon |
| chr9 | 58094123 | 58094138 | TAATGCTGTGT_A_GGAG | C | Ccdc33 | Intron |
| chr8 | 128989362 | 128989377 | TAATGCTG_GTC_TGGAG | C | Ccdc7 | Intron |
| chr8 | 118368810 | 118368827 | TAgATGCTGTG_CATGGAG | C | Cdh13 | Intron |
| chr2 | 35123117 | 35123133 | TA_TCCTGTGTCATGGAG | C | Cep110 | Intron |
| chr2 | 69045282 | 69045298 | TAT_GCTGTGTCATGAAG | C | Cers6 | Intron |
| chr6 | 89429442 | 89429458 | TAATGCTGTGT_GTGGAG | C | Chchd6 | Intron |
| chr16 | 10635188 | 10635206 | TAATGCTGTGTTtATGGAG | C | Clec16a | Intron |
| chr6 | 123140308 | 123140324 | TAATACTGTGTCATGGAA | C | Clec4a2 | Intron |
| chr9 | 97933327 | 97933343 | TAATGCTGT_TGATGGAG | C | Clstn2 | Intron |
| chr6 | 102457003 | 102457020 | TGATCCTGTGTCATGGAG | C | Cntn3 | Intron |
| chr6 | 46280099 | 46280116 | TTGTGCTGTGTCATGGAG | C | Cntnap2 | Intron |
| chr12 | 101996702 | 101996718 | TAATGTTGTGTCATGGAA | C | Cpsf2 | Intron |
| chr8 | 15953641 | 15953658 | TGATGCTGTGTCCTGGAG | C | Csmd1 | Intron |
| chr8 | 16603199 | 16603215 | TTATGCTG_GTCATGGAG | C | Csmd1 | Intron |
| chr10 | 63534209 | 63534226 | TCCTGCTGTGTCATGGAG | C | Ctnna3 | Intron |
| chr19 | 39408080 | 39408097 | TAATGATGTCTCATGGAG | C | Cyp2c38 | Intron |
| chr19 | 39253765 | 39253782 | TAATGCTGaTGT_ATGGAG | N | Cyp2c53-ps | - |
| chr5 | 145855833 | 145855848 | TAATG_T_TGTCATGGAG | C | Cyp3a11 | Intron |
| chr5 | 145775159 | 145775174 | TAATG_T_TGTCATGGAG | C | Cyp3a44 | Intron |
| chr2 | 104259095 | 104259112 | TCATGCTGTGTCAAGGAG | C | D430041D05Rik | Intron |
| chr12 | 38565512 | 38565528 | TGA_GCTGTGTCATGGAG | C | Dgkb | Intron |
| chr8 | 36941457 | 36941472 | TAATG_TGT_TCATGGAG | C | Dlc1 | Intron |
| chr19 | 25514017 | 25514035 | TAATTCTGTGTaCATGGAG | C | Dmrt1 | Intron |
| chr9 | 21834525 | 21834541 | TAA_GCTGTGTGATGGAG | C | Dock6 | Intron |
| chr14 | 41079034 | 41079049 | TAATGCT_T_TCATGGAG | C | Dydc1 | Intron |
| chr12 | 99832087 | 99832104 | TAATGCAGTGTGATGGAG | C | Efcab11 | Intron |
| chr18 | 33820562 | 33820578 | TAA_GCTGTGTCAGGGAG | C | Epb4.1l4a | Intron |
| chr16 | 59963492 | 59963507 | TAATG_TGTGT_ATGGAG | C | Epha6 | Intron |
| chr6 | 33600370 | 33600385 | TAATGT_GTGTC_TGGAG | C | Exoc4 | Intron |
| chr9 | 76523386 | 76523401 | TAATGCT_TG_CATGGAG | C | Fam83b | Intron |
| chr2 | 62509862 | 62509878 | TA_TGCTGTGTCCTGGAG | C | Fap | Intron |
| chr4 | 95772152 | 95772168 | TAATGCTATGTCA_GGAG | C | Fggy | Intron |
| chr1 | 174698224 | 174698240 | TAATGCTGTGGC_TGGAG | C | Fmn2 | Intron |
| chr7 | 89358818 | 89358834 | TAA_GCTGTGTCATGTAG | C | frizzled 4 | Intron |
| chr6 | 97425717 | 97425732 | TAAT_CTGTGTCATGG_G | C | Frmd4b | Intron |
| chrX | 168015938 | 168015954 | TAATGCTGTGTCAT_CAG | C | Frmpd4 | Intron |
| chr1 | 84714070 | 84714086 | TAATG_TGAGTCATGGAG | C | G530015K09 | Intron |
| chr2 | 54503830 | 54503848 | TAATGCTGaTGcCATGGAG | C | Galnt13 | Intron |
| chr14 | 23089849 | 23089865 | TAATGCTGTGT_ATTGAG | N | Gm10248 | - |
| chr4 | 12308074 | 12308092 | TAAgTGCTGTGTCATTGAG | N | Gm11846 | - |
| chrX | 155899283 | 155899299 | TAATTCTGT_TCATGGAG | C | Gm15155 | Intron |
| chr13 | 28948119 | 28948135 | TAATGCTGTGTCT_GGTG | N | Gm26735 | - |
| chr3 | 134401340 | 134401357 | TAATGCTGTGACTTGGAG | N | Gm26820 | - |
| chr3 | 118454277 | 118454294 | TAATGCTGAGACATGGAG | N | Gm26871 | - |
| chr14 | 13863753 | 13863768 | TAAT_CTGTGTCATGG_G | C | Gm281 | Intron |
| chr9 | 118532276 | 118532291 | TAATGCTGT_TCATG_AG | C | Golga4 | Intron |
| chr14 | 115541924 | 115541941 | TAATaGCTGTGTCATG_AG | C | Gpc5 | Intron |
| chr10 | 45662136 | 45662154 | TAATGCTGTGgTCATGAAG | C | Hace1 | Intron |
| chr11 | 83768063 | 83768080 | TAAT_CTGTGTCATtGGAG | C | Heatr6 | Intron |
| chr13 | 14489283 | 14489298 | TA_TGCTG_GTCATGGAG | C | Hecw1 | Intron |
| chr11 | 90374297 | 90374312 | TAA_G_TGTGTCATGGAG | C | Hlf | Intron |
| chr7 | 124086266 | 124086282 | TAAGGCT_TGTCATGGAG | C | Hs3st4 | Intron |
| chr3 | 68900070 | 68900087 | TAATGCTGTGTCtATGGAC | C | Ift80 | Intron |
| chr16 | 96390209 | 96390227 | TAATGCTGTGTtCTTGGAG | C | Igsf5 | Intron |
| chr1 | 37304581 | 37304598 | TCATGCTGTGTCGTGGAG | C | Inpp4a | Intron |
| chr6 | 148799008 | 148799024 | AAATGCTGTGTCAT_GAG | C | Ipo8 | Intron |
| chr8 | 126591262 | 126591278 | TAATGCTTTGTC_TGGAG | C | Irf2bp2 | Exon |
| chr5 | 49481327 | 49481343 | TAATGCAG_GTCATGGAG | C | Kcnip4 | Intron |
| chr7 | 45817607 | 45817623 | TCA_GCTGTGTCATGGAG | C | Kcnj14 | Exon |
| chr1 | 161129250 | 161129266 | TAATGCTGTATCATGG_G | C | Klhl20 | Intron |
| chr2 | 36084956 | 36084971 | TAATG_T_TGTCATGGAG | C | Lhx6 | Intron |
| chr10 | 7608461 | 7608477 | TAATG_TGTGTCATGGAG | C | Lrp11 | Intron |
| chr2 | 40598705 | 40598720 | T_ATGCTGTG_CATGGAG | C | Lrp1b | Intron |
| chr5 | 35096836 | 35096852 | TAAGGCTGT_TCATGGAG | C | Lrpap1 | Intron |
| chr7 | 67522355 | 67522370 | TAA_G_TGTGTCATGGAG | C | Lrrc28 | Intron |
| chr15 | 91677720 | 91677735 | TAATGCT_TGTCATG_AG | C | Lrrk2 | Intron |
| chr6 | 80624304 | 80624320 | TTAAGCTGTGTCATCGAG | C | Lrrtm4 | Intron |
| chr7 | 55236490 | 55236507 | TAATTCTGTGTCATAGAG | C | Luzp2 | Intron |
| chr4 | 123540103 | 123540118 | TAATGCTGTGTCA_G_AG | C | Macf1 | Intron |
| chr5 | 20102598 | 20102614 | TACTGCTGTG_CATGGAG | C | Magi2 | Intron |
| chrX | 105986118 | 105986133 | TAATGCTGTGT__TGGAG | C | Magt1 | Intron |
| chr13 | 103165792 | 103165809 | TAATGCTGCTGTCATGG_G | C | Mast4 | Intron |
| chr13 | 30204107 | 30204124 | TGATTCTGTGTCATGGAG | C | Mboat1 | Intron |
| chr2 | 128794285 | 128794301 | TATTGCTGTGTCA_GGAG | C | Mertk | Intron |
| chr7 | 136965926 | 136965941 | TAATG_TGTGT_ATGGAG | C | Mgmt | Intron |
| chr12 | 57394173 | 57394191 | TgAATGCTGTGTCATGtGAG | C | Mipol1 | Intron |
| chr17 | 83780341 | 83780357 | TgAATG_TGTGTCATGGAG | C | Mta3 | Intron |
| chr11 | 80710752 | 80710767 | TAAT_CTGTGTCA_GGAG | C | Myo1d | Intron |
| chr18 | 74444783 | 74444801 | TAATtGCTGCGTCATGGAG | C | Myo5b | Intron |
| chr14 | 123406776 | 123406792 | TAATG_TGTGTCA_GGAG | C | Nalcn | Intron |
| chr1 | 126215796 | 126215811 | TAATG_TGtGTCATGGAG | C | Nckap5 | Intron |
| chr3 | 25586534 | 25586549 | TAAT_CTGTGTCATGGAT | C | Nlgn1 | Intron |
| chr9 | 99470823 | 99470838 | TAATGCTGTG_CATGGAA | C | Nme9 (Txndc6) | Exon |
| chr5 | 66274249 | 66274265 | TCATGCTGTGTC_TGGAG | C | Nsun7 | Intron |
| chr9 | 48362509 | 48362525 | GAATGCTGTGTC_TGGAG | C | Nxpe4 (Fam55d) | Intron |
| chr9 | 27945855 | 27945872 | TAATGCTGGGTCAGGGAG | C | Opcml | Intron |
| chr11 | 115345762 | 115345777 | TAA_GCTGTGTCA_GGAG | C | Otop3 | Intron |
| chr17 | 10539616 | 10539632 | TCATGCTGTGTCATGG_G | C | Pacrg | Intron |
| chr8 | 61834351 | 61834366 | TAAT_CTGTG_CATGGAG | C | Palld | Intron |
| chr1 | 62110788 | 62110806 | TAcAAGCTGTGTCATGGAG | C | Pard3b | Intron |
| chr10 | 108360440 | 108360455 | TAATGCTGTGTC_TGGGG | C | Pawr | Intron |
| chr5 | 57922288 | 57922305 | AAATGCTCTGTCATGGAG | C | Pcdh7 | Intron |
| chr2 | 143649455 | 143649472 | TAATGCTGTGTGCAGGAG | C | Pcsk2 | Intron |
| chr14 | 48207181 | 48207196 | TAATGCT_TGTCATGG_G | C | Peli2 | Intron |
| chrX | 150629038 | 150629055 | TATT_CTGTGTCATGGAG | C | Pfkfb1 | Intron |
| chr9 | 105660749 | 105660766 | TcAATGCTGTGGCATGGAG | C | Pik3r4 | Intron |
| chr9 | 59674432 | 59674447 | TAA_GCTGTGTCATG_AG | C | Pkm | Intron |
| chr19 | 38595764 | 38595779 | TAATGCT_T_TCATGGAG | C | Plce1 | Intron |
| chr1 | 176262116 | 176262132 | TAATGCC_TGTCATGGAG | C | Pld5 | Intron |
| chr4 | 86666391 | 86666408 | CAATGCTGTGTCACGGAG | C | Plin2 | Intron |
| chr3 | 54368435 | 54368451 | TAATGCTGAGTCATGGAC | C | Postn | Intron |
| chr18 | 42704962 | 42704979 | TAATGCTGTGTCCTGCAG | C | Ppp2r2b | Intron |
| chr5 | 36936334 | 36936350 | TACTGCTGTGTC_TGGAG | C | Ppp2r2c | Intron |
| chrX | 101608947 | 101608962 | TAATG_TGTGTCA_GGAG | C | Prrg1 | Intron |
| chr8 | 67944708 | 67944724 | TAATGCTGAGTC_TGGAG | C | Psd3 | Intron |
| chr1 | 189757179 | 189757195 | TACTGCTGTGTCATGG_G | C | Ptpn14 | Intron |
| chr2 | 90451681 | 90451696 | TAATG_T_TGTCATGGAG | C | Ptprj | Intron |
| chr2 | 132021620 | 132021635 | TAATG_TGTGTCA_GGAG | C | Rassf2 | Intron |
| chr1 | 131222499 | 131222515 | TAATGCTGTCTCAT_GAG | C | Rassf5 | Intron |
| chr5 | 28334254 | 28334271 | TAAAGCTGTGGCATGGAG | C | Rbm33 | Intron |
| chr13 | 105251886 | 105251903 | TAATGCTGTGTgCAT_GAG | C | Rnf180 | Intron |
| chr4 | 49640850 | 49640865 | TAATG_TGTGTC_TGGAG | C | Rnf20 | Intron |
| chr16 | 74148253 | 74148268 | TAATG_TGTGTCATGGAT | C | Robo2 | Intron |
| chr2 | 113184025 | 113184041 | TAATGCTGTGTCATG_TG | C | Ryr3 | Intron |
| chr1 | 51296473 | 51296489 | TAATGCTG_GTCATAGAG | C | Sdpr | Intron |
| chr10 | 127907647 | 127907663 | TAATGCTTTGTCATGGaAG | C | Sdr9c7 | Intron |
| chr11 | 117242383 | 117242400 | TTGTGCTGTGTCATGGAG | C | Sept9 | Intron |
| chr4 | 113700085 | 113700102 | TAATGCTGTGTaCATGGAT | C | Skint5 | Intron |
| chr8 | 78719002 | 78719019 | TAATCCTGTGCCATGGAG | C | Slc10a7 | Intron |
| chr2 | 165434988 | 165435004 | TAATGCTGTGTCTTGGGG | C | Slc13a3 | Intron |
| chr19 | 28883669 | 28883686 | TAAT_CTGTGTCAcTGGAG | C | Slc1a1 | Intron |
| chr3 | 101913152 | 101913167 | TAA_GCTGTG_CATGGAG | C | Slc22a15 | Intron |
| chr11 | 53953426 | 53953443 | TCATCCTGTGTCATGGAG | C | Slc22a21 | Intron |
| chr11 | 53867948 | 53867965 | TCATCCTGTGTCATGGAG | C | Slc22a5 | Intron |
| chr11 | 120108321 | 120108336 | TA_TGCTGTGTC_TGGAG | C | Slc38a10 | Intron |
| chr12 | 30336893 | 30336909 | TAATGCTGTGCCATG_AG | C | Sntg2 | Intron |
| chr16 | 11377334 | 11377351 | TCATCCTGTGTCATGGAG | C | Snx29 | Intron |
| chr1 | 69890857 | 69890873 | TA_TGCTGTGTCATAGAG | C | Spag16 | Intron |
| chr19 | 46402879 | 46402896 | TAAaTGCTGTGTCATGGAG | C | Sufu | Intron |
| chr18 | 5109542 | 5109558 | AAATGCTGTGTCATGGAA | C | Svil | Intron |
| chr10 | 86392835 | 86392851 | TAATG_TGTGTCATGGAG | C | Syn3 | Intron |
| chr18 | 14800965 | 14800980 | TAATGCTGTGT_ATGG_G | C | Taf4b | Intron |
| chr11 | 36152352 | 36152368 | AAATGCTGTGTCATGGAA | C | Tenm2 (Odz2) | Intron |
| chr1 | 51142665 | 51142682 | AAATACTGTGTCATGGAG | C | Tmeff2 | Intron |
| chr9 | 75576264 | 75576280 | TAAT_CTGTGTCATGTAG | C | Tmod2 | Intron |
| chr1 | 105839964 | 105839981 | TAATGCTGGGACATGGAG | C | Tnfrsf11a | Intron |
| chr3 | 27340712 | 27340729 | TTATCCTGTGTCATGGAG | C | Tnfsf10 | Intron |
| chr3 | 84170281 | 84170298 | TAAcTGCTGTGTCATGGGG | C | Trim2 | Intron |
| chrX | 144675328 | 144675344 | TAATGTTGT_TCATGGAG | C | Trpc5 | Intron |
| chr13 | 56881298 | 56881315 | TAA_GCTGTGTtCATGGAG | C | Trpc7 | Intron |
| chr2 | 69948899 | 69948914 | TAA_G_TGTGTCATGGAG | C | Ubr3 | Intron |
| chr9 | 73957426 | 73957443 | TTATGATGTGTCATGGAG | C | Unc13c | Intron |
| chr3 | 109481385 | 109481402 | TAATGATGTATCATGGAG | C | Vav3 | Intron |
| chr10 | 79268756 | 79268772 | TAATGCTGTGT_ATGCAG | C | Vmn2r81 | Exon |
| chr10 | 79379396 | 79379412 | TAATGCTGTGT_ATGCAG | C | Vmn2r81 | Exon |
| chr1 | 36779831 | 36779847 | TCATGCTG_GTCATGGAG | C | Zap70 | Exon |
| chr15 | 93392739 | 93392755 | TAATTCTGTGTC_TGGAG | C | Zcrb1 | Intron |
| chr17 | 30082062 | 30082079 | AAATGCTGTGTGATGGAG | C | Zfand3 (Anubl1) | Intron |
| chr6 | 116312864 | 116312881 | TAgATGCTGTGTCA_GGAG | C | Zfand4 | Intron |
| chr15 | 68093909 | 68093925 | TTATGCTGTGTC_TGGAG | C | Zfat | Intron |
| chr4 | 55066017 | 55066033 | TAATGCTGTTTCATGG_G | C | Zfp462 | Intron |
| chr8 | 23910471 | 23910487 | TATTGCTGT_TCATGGAG | C | Zmat4 | Intron |
